# The Impact of China's Carbon Market on the Decarbonization of the Coal Power Industry: An Approach Considering Long‐Term Carbon Net Expenditures

**DOI:** 10.1002/gch2.202500093

**Published:** 2025-09-03

**Authors:** Xiaowei Du, Gang Lei, Qiang Zhao, Yuewen Li, Lishen Lin, Mengyang Ye, Fubo Dai, Xiaocong Hou

**Affiliations:** ^1^ Northwest Electric Power Design Institute Co., Ltd, of China Power Engineering Consulting Group Xi'an Shaanxi 710075 China; ^2^ SinoCarbon Innovation and Investment Co., Ltd Beijing 100007 China

**Keywords:** carbon market, coal power industry, decarbonization, long‐term carbon net expenditure

## Abstract

Coal‐fired power industry is a major carbon emitter which is responsible for global warming. The carbon market is an important tool to decarbonize the coal‐fired power industry. This study extends the cited Chinese literature on how carbon net expenditures impact investments at the technology level. A metric, the long‐term carbon net expenditure, is introduced to estimate the carbon net expenditure of decarbonization projects throughout their service life. The analysis suggests that retrofitting existing coal‐fired power units for biomass co‐firing will become economically feasible from 2043, and implementing carbon capture retrofitting will be viable from 2047. Enhancing the carbon market's long‐term development path is essential to promoting these decarbonization efforts.

## Graphical abstract

The analysis framework of the impact of long‐term carbon net expenditure on the investment decisions of coal‐fired power units.

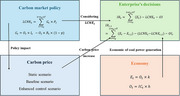

## 1 Introduction

Carbon emissions from the power sector account for ≈40% of China's total carbon emissions,^[^ ^1^ ^]^ and the emissions covered by China's National Carbon Market from the power sector reached 5.1 billion tons in 2022.^[^ ^2^ ^]^ The main source of carbon emissions from the power industry is coal‐fired power generation. In 2023, China's renewable energy power generation accounted for 33.7% and thermal power generation accounted for 66.3%, of which coal‐fired power generation accounted for more than 95%. The main means to reduce carbon emissions in the power sector include vigorously developing renewable energy, upgrading traditional thermal power generation facilities, developing biomass co‐firing and CCUS technology, optimizing grid scheduling and operation management, and improving energy use efficiency.^[^ ^1^, ^2^, ^3^, ^4^, ^5^, ^6^ ^]^ Carbon reduction in the power industry is currently encountering some major challenges. One is that there are difficulties in phasing out and upgrading coal power units, many of which were built in a relatively short period and have not yet reached their design lifespan, making it almost impossible to consider early decommission from a cost perspective.^[^ ^7^, ^8^ ^]^ Second, limited by the intermittency and volatility of power generation from renewable energy power such as wind and solar power, its large‐scale access to the grid will affect the stability and flexibility of the power system. Moreover, the current energy storage technology is still in the transition phase from R & D demonstration to commercialization, which means it is difficult to meet the demand for a high proportion of renewable energy to replace coal‐fired power due to the short time and high cost of energy storage.^[^ ^9^ ^]^ Thirdly, CCUS technology is currently costly, and the maturity of the technology needs to be improved. The cost of capturing a ton of carbon dioxide and related processing exceeds RMB 250, which is unaffordable for many companies.^[^ ^10^, ^11^, ^12^, ^13^, ^14^, ^15^ ^]^ In terms of economic considerations, whether for the construction of renewable energy power facilities, such as large‐scale solar power plants and wind farms, or for decarbonizing existing coal power plants, or for deploying CCUS technology, huge upfront investments are required. For power companies, this will undoubtedly increase their financial pressure and investment risk, which may make some companies reluctant to take action to reduce carbon emissions.

The carbon trading mechanism is a system for controlling greenhouse gas emissions, with carbon allowances or credits as the subject of market transactions. The government distributes allowances to enterprises that are covered in the system by free allocation or auction, and the enterprises make trade decisions based on their actual emissions and allowances; if they reduce emissions and have excess allowances, they can sell them for profit, and they need to purchase allowances if they emit too much.^[^ ^2^, ^3^, ^4^, ^5^ ^]^ The first carbon market in the world is the EU carbon market, launched in 2005, and has gradually matured over the years.^[^ ^16^, ^17^, ^18^, ^19^, ^20^, ^21^, ^22^, ^23^ ^]^ In recent years, the construction of carbon markets around the world has shown good momentum. As of 2023, a total of 75 countries or regions have adopted carbon pricing modes, covering a total of ≈12 billion tons of carbon dioxide, which is equivalent to 24% of global greenhouse gas emissions. Among the 75 carbon pricing initiatives implemented or planned, 36 of them are carbon market.^[^ ^24^ ^]^ China began its construction of carbon market officially in 2017 and launched it in July 2021, making it the largest carbon market in the world. After two compliance cycles of operation, the key infrastructure and key work of the National Carbon Market has withstood the full‐process closed‐loop test, proving that the carbon emissions trading system from abroad can not only run well at the pilot level in China,^[^ ^25^, ^26^, ^27^ ^]^ but also function properly after the coverage of carbon emissions rises to the national level.^[^ ^28^ ^]^ Over the past few years, Chinese enterprises have gradually accepted carbon trading as a carbon pricing tool, and their participation in it has gradually increased.^[^ ^2^, ^29^, ^30^ ^]^

However, carbon emissions from China's power sector are still on an upward trend, with a 22% increase in 2023 compared to 2018. In 2023, the newly installed capacity of thermal power was 57.93 million kWh, up 29.6% year‐on‐year. In the third quarter of 2024, a total of 145 thermal power projects were proposed, under construction or commissioned in various areas of the country. Meanwhile, zero‐carbon technologies such as CCUS and biomass power generation have yet to be promoted on a large scale. Chinese power producers are more likely to fulfill their compliance in carbon market as a temporary task, only trading on the eve of the compliance timeframe, and hardly considering the expenditure of carbon in their daily production and operation decisions, or investment decisions on new installations and unit renovation.^[^ ^31^, ^32^ ^]^ The main reason for this is that allowances in China's National Carbon Market are freely allocated, and the price is low, which makes the current compliance net expenditures borne by enterprises almost negligible compared to their operating expenditures. Moreover, China's National Carbon Market has not yet formed a long‐term roadmap, making it difficult for companies to consider carbon expenditures in long‐term decision‐making.^[^ ^33^, ^34^, ^35^, ^36^ ^]^ In fact, most companies consider the economic feasibility of thermal power plants based on the current situation of carbon prices and free allowance policies, and come to the conclusion that there is still profit.

In this paper, we introduce a key metric: long‐term carbon net expenditure in the economic analysis of coal‐fired power units investment. Long‐term carbon net expenditure is defined as a carbon net expenditure over the service life of a thermal power facility, i.e., it consider the average annual carbon net expenditure over the 20 years after commissioning. The concept of long‐term carbon net expenditure differs from social carbon cost or opportunity cost. The social carbon cost or opportunity cost reflects the cost of reducing carbon emissions at a certain point in time,^[^ ^37^, ^38^ ^]^ while long‐term carbon net expenditure reflects the net expenditure of a company on carbon allowances during a period of time.^[^ ^39^, ^40^ ^]^ The social carbon cost or opportunity cost is obtained by multiplying the carbon price by the carbon emissions, while the long‐term carbon net expenditure is obtained by multiplying the carbon price by the carbon emissions minus the free allowances. For a thermal power unit, if the number of free carbon allowances equals its total emissions over the entire cycle, the carbon expenditure for investing in the power plant is considered zero. In this case, the investment return depends solely on the cost‐benefit analysis of power generation. However, this does not imply that the marginal carbon cost or emission reduction benefits for this thermal power unit is zero.

In the long run, the carbon price will gradually increase, and simultaneously, the auction ratio of allowances will also gradually increase. The carbon net expenditure during the service life will be significantly higher than that in the year the thermal power plant is commissioned. This also provides good reference and guidance for enterprises to measure their investment returns more effectively and avoid falling into high losses with the increase of carbon net expenditure after commissioning.

Part of the previous research has focused on short‐term carbon costs or static carbon cost analysis, which commonly utilized short‐term carbon cost indicators, such as carbon emission trading prices or carbon tax costs within specific time frames.^[^ ^5^, ^25^, ^26^, ^27^, ^33^, ^34^, ^35^, ^36^ ^]^ Another part of the previous research focused on how future carbon expenditures will affect energy system modeling. Short‐term carbon emission expenditures can reflect a company's carbon emission expenditures in the short term, they fail to adequately consider long‐term net expenditure variations and uncertainties. For projects requiring long‐term investments such as new power generation facilities and emission reduction equipment upgrades, short‐term carbon cost indicators are insufficient in fully evaluating their long‐term net expenditure impacts on enterprises. Forward looking accounting of future carbon expenditures is common in many analyses of how climate policies affect investment decisions, but there is relatively little Chinese literature discussing how long‐term carbon expenditures affect technology level investments.

In contrast, the Long‐term carbon net expenditure Index can comprehensively consider the impact of time on expenditures, aligning better with the long‐term investment and operational characteristics of the coal‐fired power industry. It aids companies in better planning long‐term decarbonization strategies, preventing overlooking long‐term net expenditure risks due to short‐term decisions. For instance, when assessing whether a coal‐fired power enterprise should invest in constructing CCUS equipment, the Long‐term carbon net expenditure Index can take into account future carbon price changes, increased quota auction proportions, and shifts in carbon market policies, thereby offering a more precise assessment of the economic feasibility of the investment project. Furthermore, the Long‐term carbon net expenditure Index can promote companies to enhance investments in technological innovation since it reflects the crucial role of technology innovation in reducing carbon emission net expenditures over the long term.

## 2 Experimental Section

**Scheme 1:**
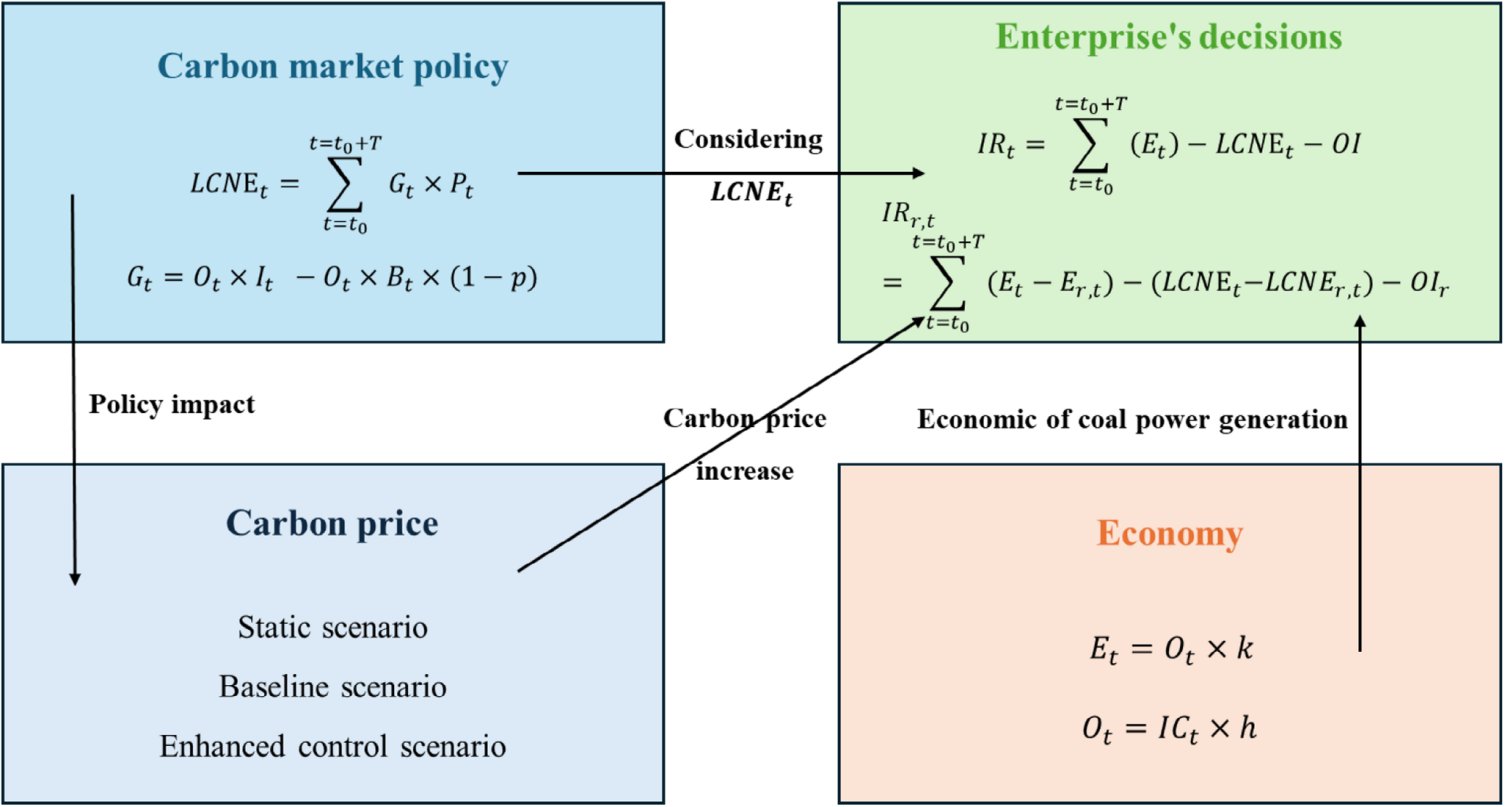
The impact of long‐term carbon net expenditure on the investment decisions of coal‐fired power units.

Long‐term carbon net expenditure is incorporated into the assessment. A highly generalized investment decision model that considers the long‐term net expenditure of carbon is shown in **Scheme** **1**. The model consists of four main systems: carbon market policy, carbon price, project technical and economic parameters, and enterprise decision‐making based on project investment return.

To calculate the net profit or net loss during the service life of commissioned coal‐fired power generation units or emission reduction and retrofit projects for commissioned coal‐fired units, the case data of coal‐fired power generation units was used, and the data related to carbon net expenditures under scenario assumptions. The calculation formula is as follows:

(1) $$LCNE_{t}=\sum_{t=t_{0}}^{t=t_{0}+T}G_{t}\times P_{t}\;\;\;\;$$

where *LCNE_t_* is the long‐term carbon net expenditures, *t*_0_ is the year of commissioning, *T* is the equipment service period, *G_t_* is the carbon allowance gap, and *P_t_* is the carbon price.

Then the investment return during the service life of coal‐fired power units were calculated and the investment return during the service life of emission reduction equipment according to the following formula:

(2) $$IR_{t}=\sum_{t=t_{0}}^{t=t_{0}+T}\left(E_{t}\right)-LCNE_{t}-OI$$

(3) $$IR_{r,t}=\sum_{t=t_{0}}^{t=t_{0}+T}\left(E_{t}-E_{r,t}\right)-\left(LCNE_{t}-LCNE_{r,t}\right)-OI_{r}$$

where *IR_t_* is the investment return during the service life of the coal power generation unit. *E_t_* is the earnings from power generation. *OI* is the original investment, refers to the capital expenditure (CAPEX), representing a one‐time upfront expenditure for purchasing emission‐generating or emission‐reduction facilities. To maintain analytical simplicity, this model does not separately account for enterprise financing arrangements or associated production financing net expenditures. *IR*_*r*,*t*_ is the investment return during the service life of emission reduction facilities. *OI_r_* is the original investment of emission reduction facilities (See **Table** **1** for details).

**Table 1:** The original investment and carbon performance.

/	Original investment	Carbon performance
Coal‐fired plant (600MW)	RMB 20 billion	Carbon intensity as industry average which is 0.7982 tCO_2_/MWh in 2023
Biomass blending retrofit	RMB 1 billion	Reducing 30% emissions by co‐firing, fuel cost increase RMB 0.06/kWh
Carbon capture retrofit	RMB 2 billion	Reducing emissions by carbon capture, cost of RMB 280/ton

Calculating the *LCNE_t_*. It is estimated according to the following equation:

(4) $$G_{t}=C_{t}-A_{t}$$

(5) $$C_{t}=O_{t}\times I_{t}$$

(6) $$A_{t}=O_{t}\times B_{t}\times \left(1-p\right)$$

where *C_t_* is the carbon emissions of coal power generation unit in period t. *A_t_* is the free allowance allocated by the government. *O_t_* is the electricity output. *I_t_* is the carbon emission intensity of power generation units. *B_t_* is the free allocation benchmark value for coal power generation. *p* is the proportion of paid allocation of allowance, that is, the auction ratio.

In this work, the profit generated by coal‐fired power generation is also calculated and compared with the net expenditure of carbon. The operating parameters and economic data of coal‐fired power generation units in this study were derived from industry cases, primarily sourced from a comprehensive survey conducted by a Chinese thermal power company.^[^ ^41^ ^]^ To enhance the robustness of the dataset, these findings were cross‐validated with multiple industry reports on thermal power and annual financial disclosures from listed Chinese thermal power enterprises, ensuring the overall reasonableness of the adopted metrics.

(7) $$E_{t}=O_{t}\times k$$

where *k* is the profit per kilowatt‐hour for coal power generation. The model assumes a constant electricity sales price RMB 0.40/kWh throughout the analysis period. The electricity generation cost set as RMB 0.33/kWh including fuel costs, labor costs, and operation, maintenance, and repair (OMR) costs (excluding depreciation, as it is already accounted for in the initial investment cost). Among these, fuel costs constitute the largest proportion, accounting for ≈75%–80% of the total cost. Labor costs represent 10%–15%, while OMR costs make up the remaining 10%.^[^ ^53^ ^]^ Consequently, the profit *k* is calculated as the difference between sales price and generation cost, yielding RMB 0.07/kWh. This simplification serves two purposes: 1) it adopts current‐year constant prices to simplify the treatment of inflationary effects, and 2) it acknowledges the practical difficulty of predicting actual price fluctuations, which typically vary within a certain range. From a long‐term average perspective, this article considers the exclusion of short‐term price volatility to be a reasonable approximation.

**Figure 1:**
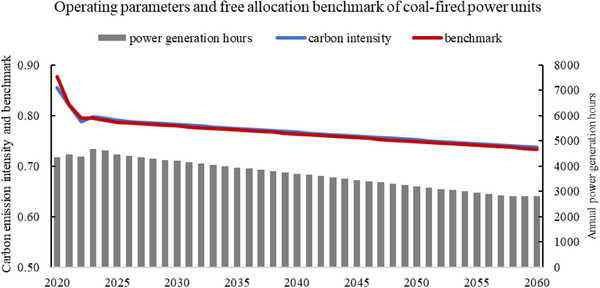
Changes in power generation hours, carbon intensity and free allocation benchmark of coal‐fired power units in each scenario.

The gross profit earned by coal‐fired power producers can be simplified as the product of profit per kWh and power generation amount, calculated via the following equation.

(8) $$O_{t}=IC_{t}\times h$$

where *IC_t_* is the installed capacity, assumed to be 600 MW. *h* is the annual power generation hours. The model assumes that with increasing installed capacity of renewable energy,^[^ ^42^ ^]^ these sources will progressively assume more baseload responsibilities, while thermal power plants will shift toward peak‐load regulation.^[^ ^43^ ^]^ The annual operating hours of thermal power generation are projected to decrease from 4628 h in 2024 to 2800 h in 2060 (See **Figure** **1**).

The calculation of the carbon net expenditure for a certain year is obtained by multiplying the carbon allowance gap by the carbon price of that year. The carbon allowance gap is the difference between the carbon emissions and the free allowances. The free allowances are jointly determined by the electricity output, benchmark value for coal‐fired units, and the auction ratio. In 2023, the average carbon intensity of coal‐fired power units with an installed capacity of 300 MW and above in China was 0.7982 tCO_2_/MWh, while the government‐set benchmark for free allowance allocation under the carbon market policy was slightly lower at 0.7950 tCO_2_/MWh (a 0.4% reduction). The model assumes the benchmark value maintained close to the average carbon intensity. Assuming that carbon emission intensity decreases slightly with technological progress, the benchmark will also decrease synchronously (See Figure 1). Under free allocation, the allowance purchased by the enterprise is sold by other enterprises. The carbon net expenditures are balanced at the entire carbon market.^[^ ^44^ ^]^ Thus, until the initial allocation of allowance through auctions, the net carbon expenditure of enterprises began to significantly increase.

The long‐term carbon net expenditure refers to the sum of the carbon net expenditures of a coal‐fired unit over its service life, that is, the accumulation of the annual carbon net expenditures during its 20‐year service period. Due to the increase in carbon prices and auction ratios, carbon net expenditures are rising year by year. The later a coal‐fired unit is put into production, the higher its long‐term carbon net expenditure will be. After calculating the long‐term carbon net expenditures for different years, they can be applied in scenario analysis to obtain the in‐service profits of coal‐fired units, and further derive the benefits of biomass co‐firing retrofit and carbon capture retrofit for coal‐fired generating units under different scenarios.

## 3 Results and Discussion

### 3.1 Scenario Analysis

Since the future carbon price will affect the current investment decision, it is necessary to select the appropriate carbon price level for the scenario analysis. The static scenario assumes that the actual situation of China's National Carbon Market in 2024 will continue, i.e., the carbon price is at the level of RMB 100/ton, and the auction ratio is zero. However, based on the policy planning of China's carbon market, it supposed to introduce auctions in the future, and the carbon price will still have a large space for increase. Therefore, when companies still follow the static scenario to analyze, it will cause significant bias to the analysis of carbon emission reduction investment.

The baseline scenario expects the national carbon price to rise steadily in the medium to long term. The main reference is the survey of 465 carbon market related industry professionals in the report “2022 China Carbon Price Survey”. The average expectation of professionals for the carbon price in 2030 is RMB 130/ton, and RMB 239/ton in 2050. As the expectation of carbon market related industry professionals, it can at least be used as a reference for power generation companies when considering carbon net expenditures.

However, this average expectation is still relatively conservative. Compared with international mature carbon markets, the EU Emissions Trading System (EU ETS) has the longest history since its launch in 2005, with its carbon price reaching the level of €70/ton in 2023, equivalent to RMB 530/ton. The proportion of allowances auctioned in the power sector in the EU ETS has also risen rapidly, from free allocation in the first phase (2005–2007), to 10% auction in the second phase (2008–2012), to 100% auction after 2013 in the third phase.^[^ ^23^, ^45^ ^]^ In comparison with the carbon pricing level required to achieve the emission reduction target, according to the IPCC expert group's study, to achieve the IPCC 2°C temperature control target, the global carbon price needs to reach $315/ton in 2050 and $578/ton in 2060. In order to achieve the IPCC 1.5°C temperature control target, the global carbon price level needs to reach at least RMB 1,715/ton in 2050 and RMB 2,328/ton in 2060.^[^ ^46^, ^47^ ^]^ According to a study by Zhang Xiliang's team at Tsinghua University, to achieve a carbon peak in 2030 and carbon neutrality in 2060, the carbon price of China would need to reach RMB 435/ton in 2040, RMB 751/ton in 2050, and RMB 2,732/ton in 2060.^[^ ^48^ ^]^ Accordingly, a higher level of carbon price is set in the enhanced control scenario, which raises to RMB 200/ton in 2030, to have a substantial impact on investment in carbon emission reduction, and RMB 578/ton in 2060, which would at least achieve the minimum theoretical level required to achieve the IPCC's 2°C temperature control target.

The baseline scenario and the enhanced control scenario demonstrate a wide range of possibilities for the future development of carbon prices. The differences in the trend and growth rate of carbon prices under different scenarios reflect the complex impacts of policy regulation, market mechanism and international factors on carbon prices. These scenario analyses have important reference value for carbon emission reduction of power generation enterprises, which can formulate more scientific and reasonable carbon emission reduction and enterprise transformation strategies based on the long‐term carbon net expenditures of different scenarios. At the same time, they are also informative for carbon market policy makers. Through the scenario analysis, it can be understood to what extent clarifying the roadmap for the medium‐ and long‐term development of the carbon market, ensuring the unified and coordinated control scope, and improving the accounting method of carbon emissions from generating units will affect the carbon emission reduction decisions of power generation enterprises.

### 3.2 Economic Analysis Taken Long‐Term Carbon Net Expenditures in Consideration

Without considering the net expenditure of carbon or with a very low net expenditure of carbon, new coal‐fired generation units have a positive return in the current and foreseeable future. Currently, when Chinese power generation enterprises do feasibility studies on coal‐fired power unit investments, they often use static scenarios for the calculation. This approach assumes that carbon net expenditures will be paid based on the current carbon price and free allocation amounts throughout the plant's economic lifetime, concluding that the investment maintains a positive internal rate of return (IRR) exceeding the benchmark rate (typically 8%),^[^ ^49^ ^]^ thereby justifying new plant construction.

However, coal‐fired units have a service cycle of ≈20 years after commissioning, and if the long‐term carbon net expenditure is considered, the carbon net expenditure will increase significantly with the future growth in carbon price and the increase in the proportion of carbon allowances allocated in a reimbursable manner. In the long term, the benefits of commissioning new coal‐fired plants will be greatly reduced, or even transformed from making a profit to incurring losses (see **Table** **2**).

**Table 2:** The turning year when various technologies become economically viable under different scenarios.

/	Newly commissioned coal‐fired power generation become uneconomical	Biomass co‐firing becomes economically viable	Carbon capture becomes economically viable
static scenario	Never	Never	Never
baseline scenario	2027	2043	2047
enhanced control scenario	2021	2028	2030

**Figure 2:**
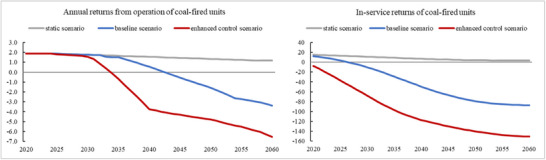
Annual and in‐service profits of coal‐fired generating units under different scenarios.

In the baseline scenario for the development of China's National Carbon Market, the operating revenues of coal‐fired units will decline significantly from 2036 onward and start to go negative from 2042 onward. Investing in coal‐fired units becomes uneconomical from 2027 onward, considering the long‐term net expenditure of carbon. In the scenario of enhanced control, the operating revenues of coal‐fired units decline significantly from 2034 onward and start to become negative from 2036 onward. Units commissioned after 2021 with long‐term carbon net expenditure face a net loss in the full‐service life (see **Figure** **2**). The article adopts a reasonable simplification by defining “uneconomical” as projects with negative annual returns during construction and operation (i.e., negative IRR), which would typically indicate an economically irrational investment under conventional financial analysis. This practical definition serves as a clear threshold for basic profitability assessment. In practice, however, enterprises operating under carbon reduction policies must consider broader factors beyond pure financial returns, including social benefits, corporate sustainability goals, and regulatory compliance. This economic criterion remains a valid and useful benchmark, while actual investment decisions may incorporate additional strategic considerations.

The economics of biomass co‐firing and retrofitting carbon capture facilities for coal‐fired units improve significantly when long‐term carbon net expenditures are considered.

**Figure 3:**
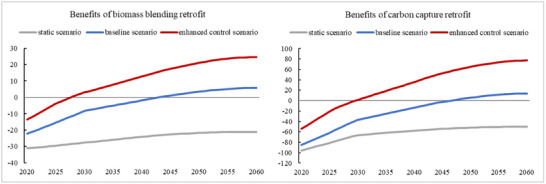
Benefits of biomass co‐firing retrofit and carbon capture retrofit for coal‐fired generating units under different scenarios.

Carbon market incentives for biomass power generation are essential for promoting this carbon‐neutral energy solution, which effectively utilizes agricultural and forestry waste while mitigating open burning pollution. Such market‐based approaches not only support stable renewable electricity generation but also foster rural development, thereby advancing China's dual carbon goals through economically viable decarbonization pathways. However, in the absence of carbon price incentives, biomass co‐firing is currently very uneconomical.^[^ ^50^, ^51^, ^52^, ^53^ ^]^ For example, if a 30% co‐firing ratio is used in the coupling scheme with a stand‐alone biomass boiler, the cost of power generation will increase by RMB 0.06 /kWh due to higher biomass fuel costs, higher equipment maintenance costs, lower combustion efficiency, and higher monitoring and management costs. China's carbon market currently does not include biomass co‐fired power generation units with a biomass co‐firing proportion exceeding 10%, meaning that such units do not benefit from the carbon expenditure savings. Because of this, the proportion of biomass co‐fired power generation units in China is currently very low. Although the National Development and Reform Commission has issued the “Action Plan for Coal Power Low Carbon Transformation and Construction (2024–2027)”, encouraging biomass use with a co‐firing ratio of over 10%, and using ultra‐long‐term special treasury bonds and other funding channels to support eligible coal power decarbonization transformation and construction. The favorable loan margin alone cannot cover the significant loss brought by the increased cost per kilowatt‐hour of biomass co‐firing. In the baseline scenario, China's National Carbon Market is expected to shift from carbon intensity control to carbon cap and trade from 2030, when biomass‐fired generating units will also be covered. Due to the carbon cycling characteristics of biomass, China's carbon accounting guidelines stipulate that the portion of biomass co‐firing will not be included in carbon emissions. This will lead to substantial savings in carbon net expenditures for coal‐fired units, which can cover the net expenditures of biomass co‐firing retrofits after 2043. Under the scenario of enhanced control, biomass co‐firing will be economically viable starting from 2028 (see **Figure** **3**).

CCUS technology has also not been widely promoted in China due to high operation net expenditures, with the current cost of carbon dioxide capture at RMB 250 per ton.^[^ ^13^, ^54^ ^]^ However, under the current verification standard of China's National Carbon Market, carbon emissions from carbon capture are not deducted, and the installation of carbon capture equipment does not change the company's amount of carbon emissions in any way. This results in the CCUS program being entirely seen as a net expenditure item at present, with only pilot projects for technical reserve purposes and no large‐scale rollout. Under the baseline scenario, with the progress of China's carbon market policies and the update of carbon emission accounting methods, it is assumed that the captured carbon dioxide can be deducted after 2030. Coupled with the gradual increase in the carbon price to exceed the cost of carbon capture, CCUS (Carbon Capture, Utilization and Storage) will become economically viable after 2047. Under the enhanced control scenario, it is assumed that the captured carbon dioxide can be deducted after 2025, and with the carbon price rising rapidly to the range of RMB 200‐300/ton under the impetus of policies, CCUS will become economically viable after 2030 (see Figure 3).

**Figure 4:**
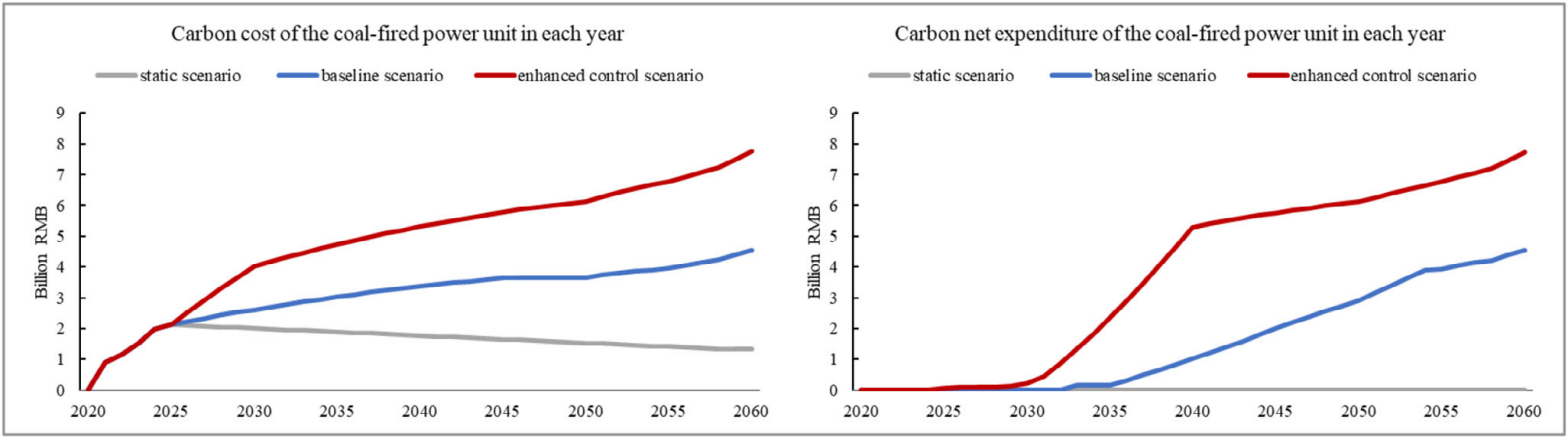
Changes in carbon cost and net expenditure in each year under different scenarios.

Currently, China's carbon market is implementing an output‐based benchmarking and intensity‐controlled approach. Carbon allowances are allocated free of charge according to the benchmark method.^[^ ^44^, ^55^ ^]^ The benchmark for power generation by coal‐fired units with an installed capacity of 300MW and above in 2023 is 0.795 tCO_2_/MWh, which is only 0.4% lower than the average carbon intensity of power generation by such units. With a carbon market price of RMB 100/ton and no auction in 2023, the actual carbon net expenditure borne by a typical coal‐fired units is only RMB 0.006 billion as shown in **Figure** **4**.

The key to promoting the implementation of emission reduction technologies by enterprises through the carbon market is to clarify policy expectations. That is because the commissioning of a generating unit and the implementation of an emission reduction technology affects the cash flow for 10 to 20 years. With clear policy expectations, the carbon pricing mechanism can be channeled into the long‐term investment decisions of enterprises. China has already issued the Interim Regulations on the Administration of Carbon Emission Trading in January 2024 as the top‐level design document for the carbon market, which stipulates that “the combination of free and paid allocation methods will be gradually implemented in accordance with relevant national requirements”. However, it has not issued a medium‐ and long‐term roadmap for carbon market construction, nor has it clarified the ratio of future allowance allocation, nor has it set up a mechanism to regulate the supply, demand and price of the carbon market. Therefore, most enterprises do not understand the future auction ratio of the carbon market, nor do they understand the future price trend, and the impact of the carbon market on future carbon emissions and carbon reduction project investment cannot be quantitatively measured. As a result, most power generation enterprises in China can only follow the traditional way of economic calculation based on a static scenario when making investment calculations. Once the auction ratio is clarified, companies will be able to better measure the economics of emission reduction projects and significantly accelerate the development and implementation of carbon reduction projects.

**Figure 5:**
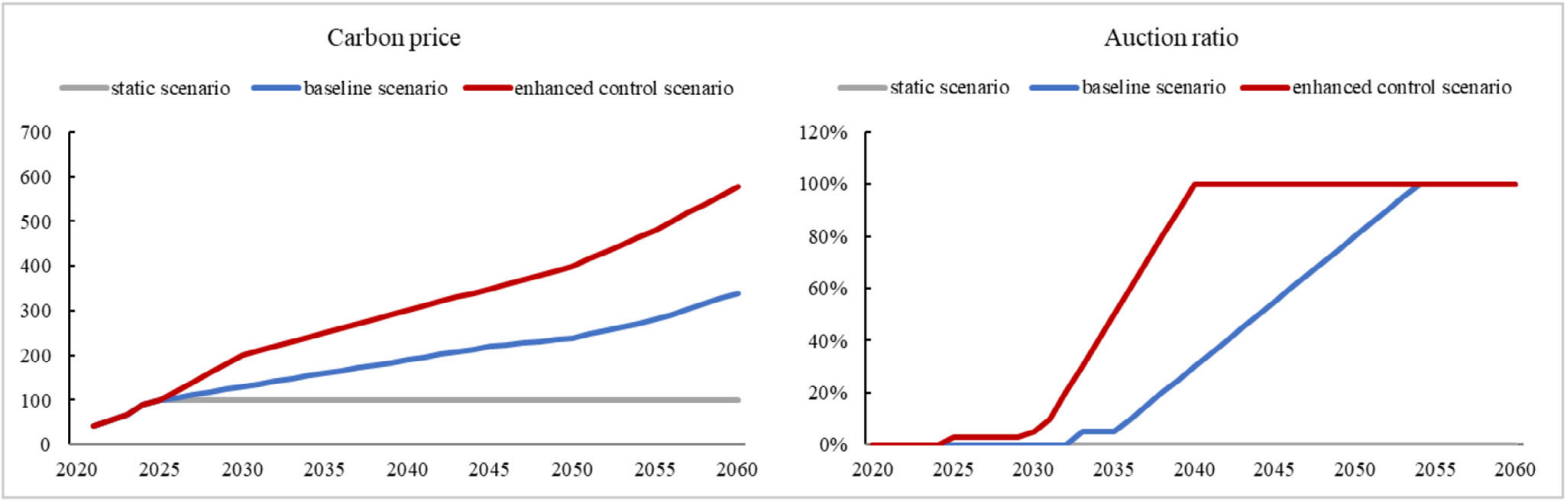
Changes in carbon price and allowance auction ratio under different scenarios.

In the baseline scenario, it is expected that the auction of allowances will be carried out by 2035, and the auction ratio will not be too high at the initial stage, which is ≈5% based on the prior experience of China's pilot carbon markets. The auction ratio will then gradually increase, reaching 100% auction of allowances for the power generation sector by 2055. The cost of carbon emissions borne by coal‐fired generating units will gradually increase after 2035, reaching more than RMB 200/ton by 2048 (see **Table** **3** and **Figure** **5** for full details).

**Table 3:** Auction ratios and carbon allowance prices under different scenarios.

/	2025	2030	2035	2040	2045	2050	2055	2060
static scenario	0%	0%	0%	0%	0%	0%	0%	0%
	100	100	100	100	100	100	100	100
baseline scenario	0%	0%	5%	30%	55%	80%	100%	100%
	100	130	160	190	220	239	280	340
enhanced control scenario	3%	5%	50%	100%	100%	100%	100%	100%
	100	200	250	300	350	400	480	578

Under the enhanced control scenario, in order to accelerate the realization of China's NDC in carbon emission reduction, China accelerates the construction of the carbon market, gives full play to the decisive role of the market in the allocation of carbon emission resources, implements the responsibility of the main bodies for emission reduction, and implements the control of the total amount of carbon emissions at an earlier stage. Allowance auctions will be introduced from 2025, with an initial auction ratio of 3%, and the auction ratio for the thermal power sector will be increased to 50% in 2035 and 100% in 2040 (see Table 3 and Figure 5 for full details).

**Figure 6:**
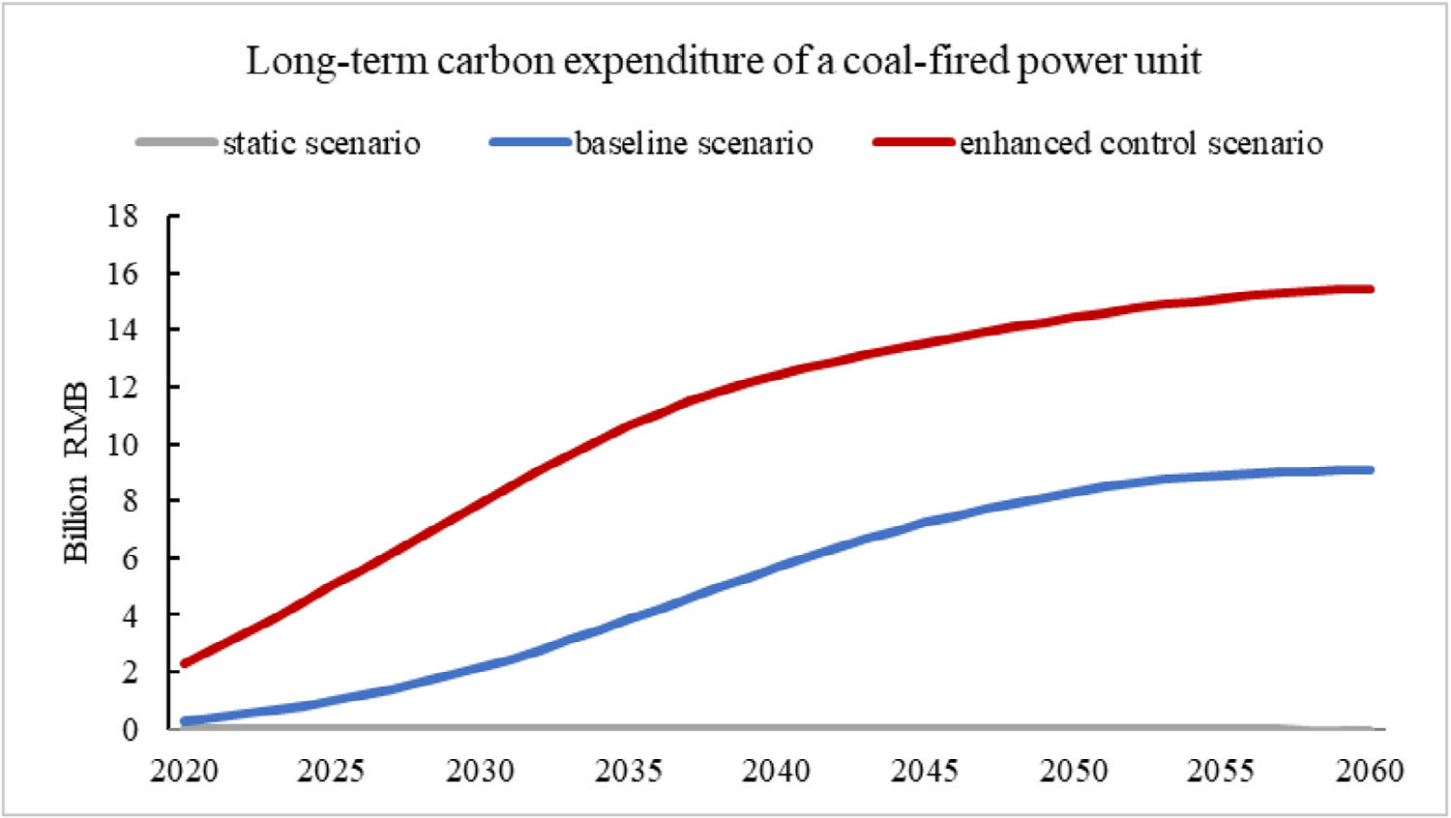
Long‐term carbon net expenditure of a typical coal‐fired power unit in different years of commissioning.

Under the enhanced control scenario, the cost of carbon emissions borne by coal‐fired generating units will increase rapidly after 2030, reaching RMB 300/ton by 2040, which will then be comparable to the cost of most abatement technologies such as biomass co‐firing or CCUS. For a typical coal‐fired power unit, the long‐term carbon net expenditure will significantly exceed the installation expenditure (≈2 billion RMB) as shown in **Figure** **6**, and become the key to promoting the decarbonization of the power generation industry.

### 3.3 Key Challenges and Policy Implications

Overall, to promote carbon emission reduction in China's thermal power generation sector, the following points are key.

First, it is important to clarify the roadmap for the medium‐ and long‐term development of China's National Carbon Market, so that companies can better define the future auction proportion of allowance for the power generation sector, the medium‐ and long‐term guidance price of carbon, and other key parameters. This will enable enterprises to better carry out the economic calculation of new unit commissioning and emission reduction and transformation of existing units during the service period, and significantly promote the transition to renewable energy in the power industry, as well as the R&D and implementation progress of CCUS, biomass co‐firing and other carbon emission reduction projects.

Second, it is necessary to incentivize biomass‐fired generation through the carbon market. There are two crucial ways, one is to include biomass co‐firing units in the carbon market, while biomass combustion is not counted as carbon emission resource; the second is to exclude biomass co‐firing units from the carbon market, while allowing biomass combustion units to develop CCER projects. This can effectively encourage enterprises to apply biomass co‐firing technology more widely for carbon reduction.

Third, the key to motivating thermal power units to implement CCUS through the carbon market is to deduct the carbon emissions from CCUS from the verified carbon emissions of power plants, subject to certain technical conditions. In this way, when the carbon price exceeds or is close to the cost of CCUS, it can effectively incentivize power producers to apply CCUS technology widely.

It is important to note that this study has several important limitations. First, there is still a great deal of uncertainty in the carbon market policy. Second, the expenditure of carbon only considers the environmental impacts of carbon and ignores the environmental influence of other pollutants, the environmental benefits of green electricity certificates, and the capacity tariff of thermal power. Third, it does not consider the synergistic effects of other carbon reduction policies within China's broader policy mix, including carbon market, renewable quotas, green power trading, subsidies, and efficiency standards, which collectively drive emission cuts by mandating clean energy adoption and lowering investment risks, thus shaping power firms' decarbonization strategies and warranting deeper research on their optimal design. Finally, the scenario assumes that the carbon net expenditure is rising in the long‐term trend, but there may be large fluctuations in the short‐term and a period of low carbon prices. The deterministic method used in this article is a methodological limitation, as it does not explicitly account for price fluctuations or policy uncertainty. This simplification may lead to an overestimation of investment stability in low‐carbon technologies, particularly for projects sensitive to short‐term carbon price variations (e.g., coal‐to‐biomass conversions). However, the core conclusions about long‐term decarbonization pathways remain valid, as they align with the fundamental premise of rising carbon net expenditures under climate policy frameworks. Future research could enhance robustness by incorporating stochastic carbon price modeling.

## 4 Conclusion

This study innovatively introduces a Long‐term carbon net expenditure Index to investigate the impact of the Chinese carbon market on decarbonization in the coal‐fired power industry. This index surpasses the limitations of short‐term or static carbon cost analyses by considering factors such as carbon price fluctuations within the service life of coal‐fired facilities, changes in quota allocation policies. It provides comprehensive and long‐term operational expenditure references for enterprises to formulate decarbonization strategies and investment decisions, enhancing decision‐making scientific and foresight.

Without considering long‐term carbon net expenditures, newly commissioned coal‐fired units may seem profitable; however, biomass co‐firing or carbon capture is uneconomical. When factor in long‐term carbon net expenditures, under the baseline scenario, new coal‐fired units will face losses from 2027. Retrofit for biomass co‐firing of existing units will be economically viable from 2043, and carbon capture retrofit from 2047. In the enhanced control scenario, biomass co‐firing will be viable from 2028, and carbon capture from 2030. For coal‐fired power enterprises, the Long‐term carbon net expenditure Model aids in accurately evaluating investment projects. This study also serves as a decision‐making reference for policymakers, assisting in determining a reasonable timeline for increasing auction proportions and carbon price regulation targets, thus enhancing the guiding role of the carbon market in decarbonizing the coal‐fired power industry.

## Conflict of Interest

The authors declare no conflict of interest.

## Data Availability

Research data are not shared.
